# Supplementary material for: Prevalence of and risk factors for depression, anxiety, and stress in non-hospitalized asymptomatic and mild COVID-19 patients in East Java province, Indonesia
Source: PLoS One. 2022 Jul 7;17(7):e0270966. doi: 10.1371/journal.pone.0270966 (PMC9262201; doi:10.1371/journal.pone.0270966)
Supplement: Attachment — Submitted filename: Response to reviewer.docx [file pone.0270966.s002.docx]

Manuscript ID	: PONE-D-21-21429

Manuscript Title	: Prevalence of and risk factors for depression, anxiety, and stress in confirmed non-severe COVID-19 patients in East Java province, Indonesia

Academic editor:

| **No.** | **Academic Editor comments** | **Response to Academic Editor** |
| --- | --- | --- |
| 1 | My general impression is that a lot  of work needs to be done to address the comments of the reviewers and toaddress my comments. I would like to advise you strongly to ask a native speaker to check/correct the revised manuscript (the language errors distract too much). | Dear Academic Editor,  Thank you for the comment. We have already asked reputable language editing service to do a final check for the revised manuscript. |
| 2 | Your introduction lacks, given your research question, a clear overview of what is (un)known about the mental health of the infected and/or people who are forced to stay in quarantine, as well as groups at risk for mental health problems. It is simply incomplete and too short. Please pay more attention towards the different periods during this pandemic. | Dear Academic Editor,  Thank you for your comment. We have improved the introduction section in the revised manuscript. |
| 3 | Please be much more critical on studies examining effects of the pandemic (very often using convenience samples and cross sectional studies without reference data prohibiting any valid conclusion about the effects of this pandemic on mental health), and include the recent meta-analysis of prospective studies of Robinson et al. 2021(doi: 10.1016/j.jad.2021.09.098). | Dear Academic Editor,  Thank you for your comment. We have added the recent meta-analysis of prospective studies of Robinson et al. in the introduction section (line 87-90) and in the discussion section (line 229-231) to highlight the variation of mental health symptoms in different periods of the pandemic. We also tried to be more critical on other studies examining effects of the pandemic throughout the revised manuscript. |
| 4 | Clarify which IRB was involved (not only the code of 201…..). | We have added the IRB that was involved in this study (line 134-135). |
| 5 | Please explain how potentially respondents were contacted to ask to participate in your study.  How were the questions administered (written, online, verbal?).  When were the questions administered (during quarantine,afterwards and how many weeks after the quarantine). Were other family members infected, in hospital because of infection, deceased following infection, etc. | Dear Academic Editor,  The potential respondents were verbally offered to participate in this study by the registrar when they first came to the quarantine facility.  The questions are written questions.  The questions are administered before quarantine, in order to avoid the possible effect of quarantine towards mental health.  We have given the explanation in the method section.  Because we want to avoid low response rate, we decided to not giving too many questions in the questionnaire. Therefore, we do not have data regarding COVID-19 infection status of respondents’ other family members. However, in the revised manuscript, we have added two additional variables, job status and history of self-quarantine. |
| 6 | No information is provided about the non-response and results of the non-response analyses. | Dear Academic Editor,  We have added the information about the non-response respondents in the result section (line 171-173) and also present the flow diagram in the fig 1. |
| 7 | Cronbachs alpha’s of the DASS-21 scales of the study sample are missing. | Dear Academic Editor,  We have added the Cronbach alpha of DASS-21 from previous study that did the adaptation to Bahasa Indonesia and validate it (line 154). |
| 8 | Explain how a history of mental illness was assessed. | Dear Academic Editor,  History of mental illness was assessed by giving a verbal question to the prospective respondents (line 128-130). |
| 9 | Explain and clarify which “independent variables” were examined (and introduce/explain all in the introduction). | Dear Academic Editor,  We have introduced the independent variables in the introduction section (line 73-76). We have also explained and clarified which independent variables were examined in the result section (line 187-188). |
| 10 | You entered the variables in the multivariate analyses that were significant in the univariate analyses on p <.025 level. I don’t understand this strategy. It is more informative to include all predictors in the multivariate analyses (and thus you can skip the univariate analyses). | Dear Academic Editor,  Thank you for your comment. We conducted regression analysis in two steps because we would like to also see and present the unadjusted odds ratio of each variable, in addition to the adjusted odds ratio. |
| 11 | Show the prevalence of anxiety, depression and stress of all predictors in the table, and clarify which cut-off’s were used in the logistic regression analyses. | Dear Academic Editor,  Thank you for the suggestion. We have presented the prevalence of anxiety, depression, and stress of all predictros in the table 1. We also clarify which cut-off were used in the logistic regression analysis in the statistical analysis sub-section of method section (line 167-168). |
| 12 | The text suggest that you only examined age, gender, marital status, having a child, and income, besides administering the DASS-21. Is this correct? If not, why did you not include these other variables? | Dear Academic Editor,  In the revised manuscript, we also added two additional variables ,which are job status and history of self-quarantine.  Because we want to avoid low response rate, we decided to not giving too many questions in the questionnaire. |
| 13 | Please improve your tables and present all relevant info, including explained variance, statistics of all include variables. | Dear Academic Editor,  Thank you for your suggestion. I have improved the tables and present all relevant info, including explained variance and statistics of all included variables in the revised manuscript. |
| 14 | As the reviewers noted, the discussion is not a discussion (for example a limitation section and comparisons withthe results of other studies on infected/quarantined are completely absent). I would like to advise you to read other COVID-19 papers published in PLOS ONE on the mental health of patients/general population, that can be used as examples. | Dear Academic Editor,  Thank you for your suggestion. We have improved the discussion section in the revised manuscript. |

Reviewer#1

| **No.** | **Reviewer’s comments** | **Response to Reviewer** |
| --- | --- | --- |
| 1 | This is an interesting study but it needs mayor revision before it can be considered again. Authors should explain that they assessed non-hospitalized COVID.19 patients during the 14 days of quareenten. It is not clear. | Dear reviewer,  Thank you for the comment. We have added an explanation that we assessed non-hospitalized non-severe (asymptomatic and mild) COVID-19 patients (line 116-117).  We would like to highlight that data collection was done before the patients undergo quarantine in the facilitated quarantine (line 122-126). |
| 2 | More data of COVID19 features is needed. Time from diagnosis is needed. Maybe anxiety and depression is not the same day one than 10 days after reclusing at home. | Dear reviewer,  Thank you for the comment. We have added additional data regarding recent history of self-quarantine.  Unfortunately, we do not have the data regarding the exact duration of the self-quarantine in majority of the respondents. We have added this shortcoming in the study limitation (line 345-348). |
| 3 | Differentiation between anxiety and depression in general population, hospitalized COVID-19 patients and long COVID patients is needed. | Dear reviewer,  Unfortunately, we did not evaluate the prevalence of depression and anxiety in general population, hospitalized COVID-19 patients, and long COVID patients in our study. We have added this shortcoming in the study limitation (line 348-351). |
| 4 | Authors have assess moddo disorders in a manner that it is not commonly investigated in the literature, which increases the relevance of the paper, but more justification and discussion is needed. | Dear reviewer,  Thank you for the comment. We have added more justification regarding the importance of this study in the introduction section, and also add extensive discussion in the revised manuscript. |
| 5 | What happened with these patients about 14 days? was any follow-up of them? | Dear reviewer,  Unfortunately, we did not do a follow-up evaluation after quarantine because of manpower shortage in this study.  However, we did a prospective study on November 2020, where we found that we found that being quarantined in quarantine facilities did not worsen the mental health status. The results of the prospective study can be found in:  <https://pubmed.ncbi.nlm.nih.gov/34819203/>. |
| 6 | The role of gender needs extensive discussion since there is available data on the liteature for that. | Dear reviewer,  Thank you for the comment. We have added extensive discussion regarding the role of gender in mental health in the revised manuscript (line 281-313). |

Reviewer #2

| **No.** | **Reviewer’s comments** | **Response to Reviewer** |
| --- | --- | --- |
|  | **General recommendation** |  |
| 1 | In my opinion, the level of English used could and should be better. There are quite many grammatical errors,suboptimal choices of verbs etc. Consider asking a native speaking colleague to copy-edit the manuscript. | Dear reviewer,  We have already asked reputable language editing service to do a final check for the revised manuscript. |
| 2 | Including the outcomes of the univariate regression models does not fulfill the criteria of making all data underlying the findings described fully available; please add a dataset containing the raw data to fulfill this criterion. | Dear reviewer,  We have uploaded the dataset containing raw data of this study as supplementary material. |
| 3 | Title  - I think “risk factor” should be “risk factors”, even though only one risk factor was identified. | Dear reviewer,  Thank you for the suggestion. We have revised the title of the manuscript. |
|  | **Abstract** |  |
| 4 | I am missing the importance of your work/knowledge gap your research is filling in in your abstract | Dear reviewer,  Thank you for the comment. We have added the importance of our work in the abstract. |
| 5 | “Study population was non-severe…” (line 31); I think “comprised” or “consisted of” would be a better verb | Dear reviewer,  Thank you for the suggestion. We have revised the words in the revised manuscript. |
| 6 | “that was” (lines 31) should be “which  were” | Dear reviewer,  Thank you for the suggestion. We have revised the words in the revised manuscript. |
| 7 | “Collected data …. version 25.0” (lines 33-34) should be omitted from the abstract. | Dear reviewer,  Thank you for the suggestion. We have omitted the sentence in the revised manuscript. |
| 8 | “There were … in this study” (lines 34-35); Out of how many patients were those 608 included, in other words what was the inclusion/response rate. | Dear reviewer,  Thank you for the comments. There were 778 patients that came to the quarantine facility during the study period. The response rate was 78%. We have added the details in the revised manuscript. (line 171-173) |
| 9 | “From 608 respondents …” (line 35); in the phrase before it was already mentioned that there were 608 participants. Iwould suggest changing this to “Of these… “ | Dear reviewer,  Thank you for the suggestion. We have changed the words according to your suggestion in the revised manuscript. |
| 10 | As you only used a screening instrument to detect psychological symptoms, I would suggest weaken the statements regarding having depression, anxiety, or stress. I think “reported” would be a better choice of verb. | Dear reviewer,  Thank you for the suggestion. We have changed the words “had depression”, “had anxiety” and “had stress” to “reported symptoms of depression”, “reported symptoms of anxiety”, and “reported symptoms of stress”. |
| 11 | would suggest adding the association between the severity of psychological symptoms (i.e., the scores) and female gender to your abstract. | Dear reviewer,  Thank you for the suggestion.  When we re-do the statistical analysis after adding two additional variables, we realized that our dataset do not fulfil the requirement for linear regression analysis. Therefore, in the revised manuscript, we omitted all results from linear regression analysis. |
| 12 | I don’t think the conclusion drawn can be extrapolated from the data presented. Although you demonstrate that female sex is a risk factor for psychological symptomatology, this does not mean that males doesn’t report any psychological symptoms (I imagine, as this comparison is not ready made in the manuscript). | Dear reviewer,  Thank you for the comment. Because of the additional two variables in the analysis, the results and the conclusion in the revised manuscript changed. |
|  | **Introduction** |  |
| 13 | I think in general the introduction is lacking important references. | Dear reviewer,  Thank you for the comment. We have improved the introduction and added more important references. |
| 14 | Lines 44 to 50 are not relevant, as everybody already knows about the COVID-19 pandemic. Try to be more creative when writing your introduction. | Dear reviewer,  Thank you for the suggestion. We have revised our introduction in the revised manuscript. |
| 15 | I think much more literature regarding recent findings about psychological distress during COVID-19 should be added and described. The introduction in its present form lacks a description of why this research is important and what are the knowledge gaps. Why would you think that patients have psychological distress during COVID-19/a SARS-CoV-2infection? What is already known? What isn’t known? What is your research going to add to the literature. I think “However, there is no study conducted that evaluates the mental health conditions among confirmed COVID-19 patients in Indonesia to this date” is not enough justification for your research. | Dear reviewer,  Thank you for the comment. We have added more literature regarding recent findings about psychological distress during COVID-19, what are the knowledge gaps, and the importance of our research in the revised manuscript. |
| 16 | Overall, I think the introduction is to general, especially the first paragraph, and lacks a clear description of already published literature concerning this subject. | Dear reviewer,  Thank you for the comments. We have improved our introduction in the revised manuscript. |
|  | **Methods** |  |
| 17 | “Study population was” (line 65): This is not correct English. Also, this sentence is too long, I would split it up in more sentences for clarity. | Dear reviewer,  Thank you for the correction. We have revised the words and split the sentence into two sentences in the revised manuscript. |
| 18 | “Inclusion criteria was … in this study” (lines 69-70): There are various grammatical errors in this sentence, please rephrase. | Dear reviewer,  Thank you for the correction. We have revised the grammatical errors in this sentence in the revised manuscript. |
| 19 | “relevant institutional reviewer board” (lines 71-72): Please include the name of the institutional review board. | Dear reviewer,  We have added the name of the institutional review board in the revised manuscript. |
| 20 | I was expecting a paper describing the validity of the used screening questionnaire for reference 8. This reference however does not study this matter, but only uses the same instrument. Please provide a reference that is indeed studying the validity of the instrument. Are there any other references about the development of the DASS-21? | Dear reviewer,  The Indonesian version of DASS-21 that we used in this study was translated and validated by the authors from the ref 8 (ref 35 in the revised manuscript). The validation process is part of the paper. The full-text of the ref 8 (or 35) can be downloaded [here](https://www.researchgate.net/publication/323807258_Depression_Anxiety_and_Stress_among_Undergraduate_Students_in_Jakarta_Examining_Scores_of_the_Depression_Anxiety_and_Stress_Scale_According_to_Origin_and_Residency).  Before using that questionnaire, we asked the corresponding authors for the detailed validity and reliability test report, to confirm whether or not we can use their Indonesian version of DASS-21 for our study.  The only official Indonesian version from the developer of DASS is for the [DASS-42](http://www2.psy.unsw.edu.au/Groups/Dass/Indonesian/Indonesian.htm). For DASS-21, there is no official Indonesian version. There is a study in 2013 that aimed to adapt the DASS-21 to Indonesian version, but it failed.  (<https://pubmed.ncbi.nlm.nih.gov/23425257/>)  Surprisingly, there are studies that cited the publication in 2013 and claimed that the DASS-21 has been successfully translated to Indonesian language. This are the two examples for your perusal (the claims can be found in the method section):   1) <https://pubmed.ncbi.nlm.nih.gov/33615018/> 2) <https://pubmed.ncbi.nlm.nih.gov/34901916/> |
| 21 | Which sociodemographic characteristics were asked for? In the results, only age, sex, marital status, having a child and incomes are listed, are those also the only demographic variables that were gathered? | Dear reviewer,  In the revised manuscript, we have added two additional variables, job status and history of quarantine. Because we want to avoid low response rate, we decided to not giving too many questions in the questionnaire. |
| 22 | I think the listing of the cut-off values is quite chaotic. Maybe it is an idea to add a table (in the main manuscript or additional files) which gives a clear oversight of the cut-of values | Dear reviewer,  Thank you for the suggestion. We have listed the cut-off values of each mental health subscale in Table 1 of the revised manuscript. |
| 23 | It is totally unclear to me why only the abovementioned variables were used to identify risk factors for the developmentof anxiety, depression, or stress. Was this based on existing literature? Please describe this in much more detail in the manuscript. | Dear reviewer,  We have introduced the variables that were used to identify the development of mental health symptoms in the revised manuscript (line 73-76). |
| 24 | Were all patients included in just one month (September 2020)? Otherwise, specify the start and ending date of thestudy. | Dear reviewer,  Yes, all patients included in just one month (From 1 Sepember to 30 September 2020). For clarity, we revised the study period from “September 2020” to “betwee 1 and 30 Setpember 2020” in the revised manuscript. |
| 25 | When and how were patients recruited and included? | Dear reviewer,  Respondents were recruited before they underwent quarantine. They were recruited when they came to the registration desk to register for quarantine. During registration, if the registrar found that the patients fulfilled the inclusion criteria, they were offered to participate in this study. We have added the details of the recruitment process in the revised manuscript (line 122-126). |
| 26 | You only describe that you use logistic and linear regression analysis, but it is not clear for which they are used. This should be made more clear. | Dear reviewer,  Thank you for the comment. We have added the explanation about the use of logistic regression analysis. |
|  | **Results** |  |
| 27 | I am missing the total number of patients who were eligible, from which the 608 participants were included. It might be an idea to include a flow diagram to show this. | Dear reviewer,  Thank you for the suggestion. We have added the information about the total number of patients who were eligible using flow diagram in Figure 1. |
| 28 | I am missing a table depicting the descriptives of the main outcomes, which are the prevalence and severity (i.e., sumscores) of the domains of the DASS-21. It is now only described in the text. In this way, you can omit the text regarding the distribution among mild, moderate or severe psychological distress, and just refer to the table. | Dear reviewer,  Thank you for the suggestion. We have added another table (table 3) depicting the prevalence and the severity of the depression, anxiety, and stress symptoms. |
| 29 | It might be an interesting idea to group the cohort based on their outcomes (i.e., patients who reported psychological stress vs. patients who  did not), and compare these groups. | Dear reviewer,  Thank you for the suggestion. Unfortunately, we did not have the DASS-21 score of the respondents after they finished their quarantine in the quarantine facility. |
| 30 | I think the first paragraph could be written in a less point-to-point manner. | Dear reviewer,  Thank you for the suggestion. We have revised the first paragraph of the result section in the revised manuscript. |
| 31 | Table S1: Where does ‘COR’ stand for? Abbreviations should be explaned for each table (not this is only done for tableS2 and Table 3. | Dear reviewer,  In the revised manuscript, Table S1 (the univariate analysis of logistic regression) is integrated with the multivariate regression analysis results (Table 4, 5, and 6). For table S2, as we mention previously in our response in the abstract section that our dataset do not fulfil the requirement for linear regression analysis, we therefore omitted it. |
| 32 | Table 2: I do not understand why ‘having at least one child’ is not added to the multiple logistic regression model, as these variables had a p-value <0.25 in the univariate logistic regression model. | Dear reviewer,  It was actually included in the analysis, but since we use backward logistic regression, we only wrote down the variables in the final model. As “having at least one child” variables was not in the final model, we therefore did not write down in the multivariate regression analysis table. |
| 33 | Tabel 2: Why does this table include ‘depression’ as no multiple regression analysis was conducted for this outcome? | Dear reviewer,  Thank you for your correction. We realized that column regarding depression should be omitted instead in the previous manuscript.  However, in the revised manuscript, after the additional of two variables, job status and history of quarantine, both variables serves as independent risk factors for depression. |
| 34 | Table 3: I would suggest adding all variables, including their Beta, SE, etc. for all components which were added to themodel. In that way, you don’t have to describe everywhere for which other variables were adjusted. | Dear reviewer,  Since we realized during re-evaluation of our statistical analysis that our dataset do not fulfil the requirement for linear regression analysis, we omitted all results from linear regression analysis. |
| 35 | Table 3: I do not understand why the multiple linear model for anxiety was adjusted for having a child and marital  status,when marital status did not have a p-value below 0.25 in the univariate analysis. | Dear reviewer,  Since we realized during re-evaluation of our statistical analysis that our dataset do not fulfil the requirement for linear regression analysis, we omitted all results from linear regression analysis. |
| 36 | Overall, I think the results section can be written in a more clear and organized manner. | Dear reviewer,  We have re-write the result section in the revised manuscript. |
| 37 | Discussion |  |
|  | I think the discussion needs a lot of work; in its present form it is 1) way too shallow and 2) the authors have failed todiscuss any truly important topics. | Dear reviewer,  Thank you for the comment. We have revised the discussion section in the revised manuscript. |
| 38 | Try to rewrite the discussion using the following paragraphs:  First paragraph: Short repetition of the main results.  Second and further paragraphs: Discussing observed main results with recent literature.  Last paragraph: Limitations  In its present form, the authors already start discussing their results in the first paragraph by mentioning another study inCOVID-19 confirmed cases. I think, if that is a point of discussion, the authors should elaborate more on these results,and also compare with, for instance, papers which report the prevalence of psychological distress after  COVID-19. Otherliterature concerning this matter is hardly referred to.  Also, the only true discussion the authors describe is the fact the women are more prone to psychiatric sequelae afterCOVID-19. That is, in my opinion, not new or surprising at all and definitely not enough on its own for a discussion. Thefact that the whole manuscript only counts 17 references, of which 5 are not even scientific papers, underscores my point. | Dear reviewer,  Thank you for the suggestion. We have incorporated your suggestion in our revised manuscript. |
|  | Conclusion |  |
| 39 | The conclusion in its present form is just a repetition of the main results of this study and not so much of an conclusion,except that the psychological well-being of COVID-19 positive women should be evaluated regularly. In my opinion, the conclusion section should never contain number, as the results section is the only place to report these. As such, I think the conclusion should be rewritten. | Dear reviewer,  Thank you for the comment. We have revised the conclusion section in the revised manuscript. |
