# Supplementary material for: Prevalence of and risk factors for depression, anxiety, and stress in non-hospitalized asymptomatic and mild COVID-19 patients in East Java province, Indonesia
Source: PLoS One. 2022 Jul 7;17(7):e0270966. doi: 10.1371/journal.pone.0270966 (PMC9262201; doi:10.1371/journal.pone.0270966)
Supplement: Attachment — Submitted filename: Response to reviewer.docx [file pone.0270966.s003.docx]

Manuscript ID	: PONE-D-21-21429R1

Manuscript Title	: Prevalence of and risk factors for depression, anxiety, and stress in non-hospitalized asymptomatic and mild COVID-19 patients in East Java province, Indonesia

Reviewer#1

| **No.** | **Reviewer’s comments** | **Response to Reviewer** |
| --- | --- | --- |
| 1 | The paper is much improved. Nevertheless, the introduction is still slightly confusing. Authors talk about mental health, but they do not clarify until the end of the introduction that they are talking of non infected patients. Authors should update current data on mental health in infected COVID-19 patients, both hospitalized and non-hospitalized. This should be clear in the introduction and use this information in the discussion | Dear reviewer,  We have revised the introduction by adding information about the mental health condition in both hospitalized and non-hospitalized COVID-19 patients (line 97-101).  We have also revised the discussion section by mentioning that we compared our findings with other studies that evaluate the mental health of non-hospitalized COVID-19 patients (line 220-221). In addition to that, we have also indicated in our study limitation that we did not compare our findings with hospitalized COVID-19 patients (line 350-353) |
| 2 | In addition, the title should use non-hospitalized patients instead of non-severe. | Dear reviewer,  Thank you for the suggestion. We have revised the title into “Prevalence of and risk factors for depression, anxiety, and stress in non-hospitalized asymptomatic and mild COVID-19 patients in East Java province, Indonesia” (line 1-2) We argue that it is necessary to mention about the severity of the COVID-19 symptoms in the title. |

Reviewer #2

| **No.** | **Reviewer’s comments** | **Response to Reviewer** |
| --- | --- | --- |
|  | **Abstract** |  |
| 1 | Could you add the coefficients of your regression models when mentioning the risk factors, as only mentioning those won’t help the reader understand the correlation/association | Dear reviewer,  Thank you for the suggestion. We have added the adjusted odds ratio and the p-value in the abstract (line 50-54). We add the p-value instead of the 95%CI due to the word limits of 300 words. |
| 2 | I would suggest adding some baseline demographics of the cohort, such as age, gender etc. to the abstract. | Dear reviewer,  Thank you for the suggestion. We have added the baseline demographics (age and sex) of the included patients in the abstract section (line 47). |
| 3 | I would rephrase or even omit your recommendation. I think it is a nice conclusion that psychological distress was not that apparent in non-severe COVID-19 patients, but I do not believe that there should indeed be paid more attention to the psychological distress of these patients as psychological distress is that scarce. It seems even comparable or just a little bit higher (anxiety) than the expected prevalence of those disorders in non-COVID-19 times. Although I understand the authors tendency to give arecommendation, this recommendation is too general in my opinion, and I’m doubting whether the government is the one to pay attention, rather than employers (as HCW were more likely to report depression for instance). | Dear reviewer,  Thank you for the suggestion. We have omitted the recommendation in the revised manuscript. |
| 4 | Although it is the authors choice, I would prefer a structured abstract over a narrative abstract, especially considering the design of your study. | Dear reviewer,  Thank you for the suggestion. We agree that the manuscript looks better when the abstract section is structured. We have made the changes in the revised manuscript. |
|  | **Introduction** |  |
| 5 | I would pay some more attention to the first paragraph. The coronavirus disease 2019 was not identified as a global pandemic, but the spread of the virus causing this disease was classified as a pandemic. The disease does not infect people, the virus does. “Various preventive public health measures have been implemented”; as most of these measures have already been omitted, I would say ‘were implemented’ (have been implemented implies that it is currently still the case). | Dear reviewer,  Thank you for the correction. We have revised the first paragraph of the introduction (line 62-67). |
| 6 | There are still some grammatical errors in the introduction, especially in the use of verbs. | Dear reviewer,  We have asked a native speaker colleague to check the grammatical errors in the introduction. |
| 7 | I would suggest bundling the reasons for conducting your research (the lack of data from LMIC, differs across time periods etc.) to one paragraph. It helps you being more concise, which would improve the introduction. | Dear reviewer,  Thank you for the suggestion. We have bundled the reasons for conducting the research into one extensive paragraph (line 85-105). |
|  | **Methods** |  |
| 8 | “… and information about informed consent was given before the consent form was signed.” I think this should be information about the study (I hope so at least) | Dear reviewer,  Yes, what we meant is information about the study. We have revised the sentence to make it clearer (line 135-137). |
| 9 | Although all possible risk factors are mentioned in the methods now, it is not really clear to me why you choose those variables. Was is based on literature concerning risk factors for psychological distress or were they chosen as they were easily gathered? Please explain in the methods. (in other words, why would you think that risk factors would be among those gathered variables?) | Dear reviewer,  We choose those variables based on literature concerning risk factors for psychological distress during the COVID-19 pandemic and because those variables were easily and commonly gathered. We have added the explanation in the method section (line 144-149) |
|  | **Results** |  |
| 10 | “For regression analysis, all sociodemographic variables from Table 1 were included, except educational background and family income, as these variables had a higher percentage of missing data”; Could you also describe in the Methods section that variables that had a certain percentage of missing data were excluded from the data analysis. | Dear reviewer,  Thank you for the suggestion. We have added the description in the method section (line 170). |
|  | **Conclusion** |  |
| 11 | Only comment is that I would rephrase your recommendation, as also stated in the abstract. | Der reviewer,  Thank you for the suggestion. Same as in the abstract section, we have also omitted the recommendation in the conclusion section. |
